# Diamond‐Unit Crisscross Mattress Suture for Double‐Row Rotator Cuff Repair: An All‐Arthroscopic Technique

**DOI:** 10.1002/atn2.70350

**Published:** 2026-11-08

**Authors:** Xiao‐He Wang, Chao Fang, Gang Yu, Ying‐Ming Wang, Qi‐Chun Zhao

**Affiliations:** ^1^ Department of Orthopedics, The First Affiliated Hospital of USTC, Division of Life Sciences and Medicine University of Science and Technology of China Hefei Anhui China

## Abstract

This article describes the diamond‐unit crisscross mattress suture technique for double‐row rotator cuff repair using an all‐arthroscopic approach. This method combines knotted fixation at the medial row and knotless fixation, offering uniform tendon footprint coverage while reducing anchor usage. The technique is straightforward to perform and does not require additional sutures. By using a single lateral‐row anchor, it improves the contact and stability between the tendon and bone. This technique is intended for selected small‐ to medium‐sized tears, particularly those with irregular or degenerative margins.

## Graphical abstract

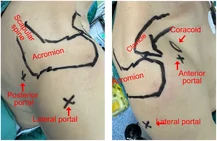

**VIDEO 1:** Diamond‐unit crisscross mattress suture technique for arthroscopic double‐row rotator cuff repair of the right shoulder. The patient is placed in the beach‐chair position, and the procedure is performed on the right shoulder. Diagnostic glenohumeral arthroscopy is first performed through the posterior viewing portal using a 30° arthroscope, followed by subacromial visualization through the lateral portal. After preparation of the rotator cuff footprint, a medial‐row anchor is inserted adjacent to the articular margin at the center of the tear. The suture limbs are passed through the supraspinatus tendon in a posterior‐to‐anterior sequence and are numbered to maintain correct strand orientation. Paired medial‐row sutures are tied to establish secure medial fixation. Three evenly distributed puncture points are then created near the free edge of the torn tendon, and the suture limbs are passed in a crisscross pattern to form interconnected diamond‐shaped mattress units. After sequential tightening, the torn tendon edge is reshaped and compressed evenly onto the footprint. Finally, all suture limbs are fixed laterally with a single knotless lateral‐row anchor to complete the repair. This video shows how the crisscross mattress configuration converts point fixation into a continuous load‐sharing construct, improving tendon‐to‐bone contact while minimizing lateral‐row anchor usage. Video content can be viewed at https://doi.org/10.1002/atn2.70350.

Rotator cuff tears are among the most common causes of shoulder pain and dysfunction, and arthroscopic repair has become the standard treatment for symptomatic full‐thickness tears.^1^, ^2^, ^3^ Despite continuous advances in suture materials and anchor technology, failure at the tendon‐bone interface remains a major concern, particularly in large or degenerative tears.^4^, ^5^ Recent studies have further emphasized that construct configuration and load‐sharing pattern, rather than anchor number alone, play a critical role in healing outcomes.^6^, ^7^, ^8^

The suture‐bridge or transosseous‐equivalent technique was developed to improve footprint contact area and initial fixation strength compared with traditional single‐row repairs.^4^ This construct has shown superior biomechanical properties and favorable clinical outcomes in multiple biomechanical and clinical studies.

However, recent arthroscopy technique reports have highlighted persistent limitations in medial‐lateral load imbalance and lateral‐row dependency.^7^, ^8^, ^9^ When knotless fixation at the medial‐row anchors is used, the overall stability of the repair largely depends on the integrity and position of the lateral anchor, as well as the precise tension of the sutures. If lateral anchor loosening or pullout occurs, the entire repair may fail catastrophically.^10^ In contrast, when knotted fixation is used, although they form a strong fixation structure and provide enhanced stability for the rotator cuff, the effective number of suture limbs crossing and covering the tendon footprint is significantly reduced. The 6 originally distributed suture limbs functionally degrade into only 3 compression points, resulting in uneven pressure distribution and potentially inadequate footprint coverage. Although using 2 lateral anchors can partially address this issue, it inevitably increases implant usage and surgical costs, which may not be necessary for small‐ to medium‐sized rotator cuff tears.^10^

In addition, conventional simple stitches may not adequately restore the native geometry of the torn tendon edge, particularly in crescent‐shaped or degenerative tears. Suboptimal alignment of the torn tendon margin may compromise tendon‐to‐bone contact and increase the risk of gap formation under cyclic loading, which has been associated with inferior healing outcomes.^1^, ^5^

To address these limitations, we describe a rotator cuff repair technique incorporating a modified mattress suture configuration at the torn tendon edge combined with single lateral‐row fixation. This technique aims to (1) combine the firm fixation brought by knotted fixation and the extensive coverage provided by knotless fixation to achieve broad and uniform coverage of the rotator cuff footprint, (2) restore a smooth and evenly tensioned tendon margin that conforms closely to the bone surface, and (3) enhance construct stability while minimizing implant usage. By applying controlled compression through a single lateral anchor, the repaired tendon can be securely and evenly seated onto the footprint.

## SURGICAL TECHNIQUE

### Patient Positioning and Arthroscopic Portal Setup

After the administration of general anesthesia and a brachial plexus block, the patient is positioned in a beach‐chair posture for the shoulder arthroscopy procedure. The trunk is raised between 45° and 60°, with the hips flexed at 40° to 50° and knees bent at 20° to 30°, ensuring the operative shoulder extends beyond the edge of the table. The head is secured in a neutral position using a foam headrest to avoid cervical hyperextension, whereas a posterior pad is positioned medially to improve glenohumeral exposure. The arm is placed in 20° of forward flexion and 40° of abduction using a hydraulic arm holder. Key bony landmarks and arthroscopic portals are identified and marked (Figure 1).

**FIGURE 1:**
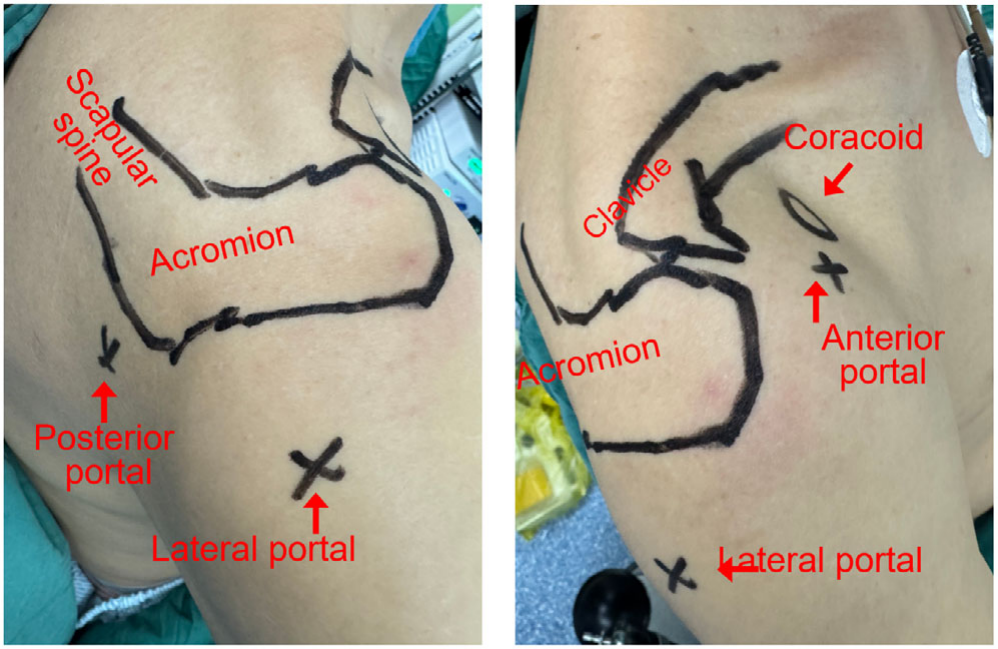
Portal mapping and patient positioning. The patient is positioned in a beach‐chair position with the right shoulder free at the edge of the operating table and the head secured in neutral alignment. Standard posterior, anterior, and lateral portals are marked using surface anatomical landmarks prior to sterile preparation. Educational message: Accurate preoperative positioning and portal planning are essential to ensure reproducible arthroscopic access and safe instrument triangulation during rotator cuff repair.

The primary posterior portal is created with a 1.5‐cm incision, 1 cm medial and 2 cm distal to the posterior‐lateral angle of the acromion. Diagnostic arthroscopy is initiated through this portal using a 30° arthroscope. For the anterior portal, needle localization is performed lateral to the coracoid apex, and the standard lateral portal is positioned 2 cm below the acromion along the supraspinatus tendon's axis. Additional accessory portals may be created as needed based on the complexity of the pathology.

### Glenohumeral Joint Evaluation and Subacromial Decompression Preparation

The glenohumeral joint is assessed through the posterior portal, followed by the anterior portal to check for damage or inflammation in the long head of the biceps tendon or subscapularis tendon (Figure 2). If a tear or SLAP lesion is found, surgery is planned based on the patient's age and activity level. If a subscapularis tendon tear is identified, fixation is performed at the lesser tuberosity. Capsular release is done if there is significant contracture.

**FIGURE 2:**
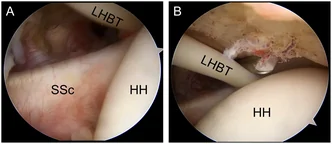
Diagnostic arthroscopy of the right shoulder. Intra‐articular evaluation is performed with a 30° arthroscope via the posterior viewing portal in a beach‐chair position. A probe introduced through the anterior working portal assesses the subscapularis tendon (A) and long head of the biceps tendon (B). Educational message: Systematic glenohumeral inspection through a posterior viewing portal is critical to identify concomitant intra‐articular pathology before proceeding to subacromial reconstruction. (HH, humeral head; LHBT, long head of biceps tendon; SS, supraspinatus.)

After intra‐articular procedures, the arthroscope is moved to the subacromial space through the posterior portal, and a lateral working portal is created. Inflammatory tissue is removed with an arthroscopic shaver and radiofrequency ablator (Smith & Nephew, Andover, MA) to expose the rotator cuff and acromion. If osteophytes or subacromial impingement are found, acromioplasty is performed with an arthroscopic burr. An anterolateral portal is created to assess the rotator cuff tear. The tendon footprint is prepared with a bone cutter, and microfracture is performed to stimulate blood flow and promote tendon‐to‐bone healing.

### Diamond‐Unit Crisscross Mattress Suture for Double‐Row Rotator Cuff Repair

The choice between a 3‐line or 2‐line anchor depends on the size of the rotator cuff tear. In this technique, a 3‐line medial‐row anchor (HEALIX ADVANCE; DePuy Mitek, a Johnson & Johnson company, Raynham, MA) is placed at a 45° angle toward the rotator cuff attachment point, near the joint margin at the center of the tear. All 6 (or 4) suture ends, originating from 3 (or 2) sutures, are sequentially passed through the rotator cuff tissue, using a Lasso suture passer (DePuy Mitek, a Johnson & Johnson company), ensuring they are positioned more than 10 mm inside the lateral edge of the tear for even distribution. Under lateral visualization, the sutures are numbered sequentially from 1 to 6, from posterior to anterior. It is essential that paired sutures (1 and 2, 3 and 4, and 5 and 6) come from the same suture (Figure 3). Sutures 1 and 2, 3 and 4, and 5 and 6 are then tied, securing the inner layer of the torn rotator cuff to the bone surface, thereby enhancing the tensile strength of the fixation (Figure 4).

**FIGURE 3:**
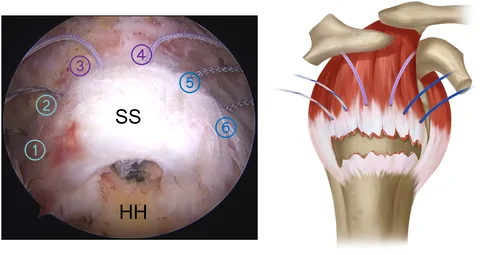
Medial‐row suture passage in crisscross mattress configuration. With the arthroscope placed in the lateral subacromial portal, a triple‐loaded medial‐row anchor is inserted at the footprint adjacent to the articular margin of the right supraspinatus tendon. Sutures are passed in a controlled posterior‐to‐anterior sequence and individually numbered to ensure correct pairing and geometric symmetry (laterality: right). Educational message: Precise suture sequencing and symmetric strand organization are fundamental for constructing a balanced crisscross mattress configuration. (HH, humeral head; SS, supraspinatus.)

**FIGURE 4:**
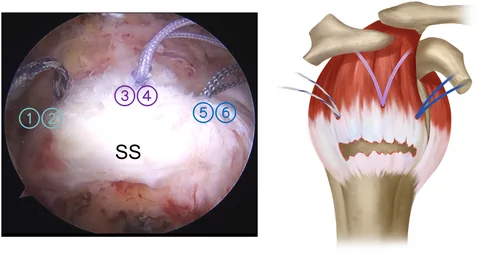
Sequential knot tying strategy. Using lateral portal visualization, paired suture limbs (1‐2, 3‐4, and 5‐6) are sequentially tied after tendon passage with the patient in beach‐chair position (laterality: right). Educational message: Structured paired knot tying ensures uniform load distribution across the tendon footprint and prevents asymmetric compression. (SS, supraspinatus.)

Next, 3 evenly distributed puncture points are made approximately 2 to 3 mm from the edge of the rotator cuff tear. The exit points should be near the tear edge, avoiding too vertical of an entry angle (Figure 5), which increases the contact area between the tear edge and the bone and prevents the edge from lifting. The sutures are passed sequentially from posterior to anterior through the 3 puncture points: 1 and 3, 2 and 5, and 4 and 6 (Figure 6). The sutures are tightened, causing the rotator cuff tear edges to tightly conform to the bone surface, with secure fixation confirmed on probing. It is important to note that tightening sutures 3 and 4 provides lateral stability by drawing the tear edges together. In the case of a 2‐line anchor, 2 puncture points are selected, and sutures 1 and 3 and 2 and 4 are passed through sequentially.

**FIGURE 5:**
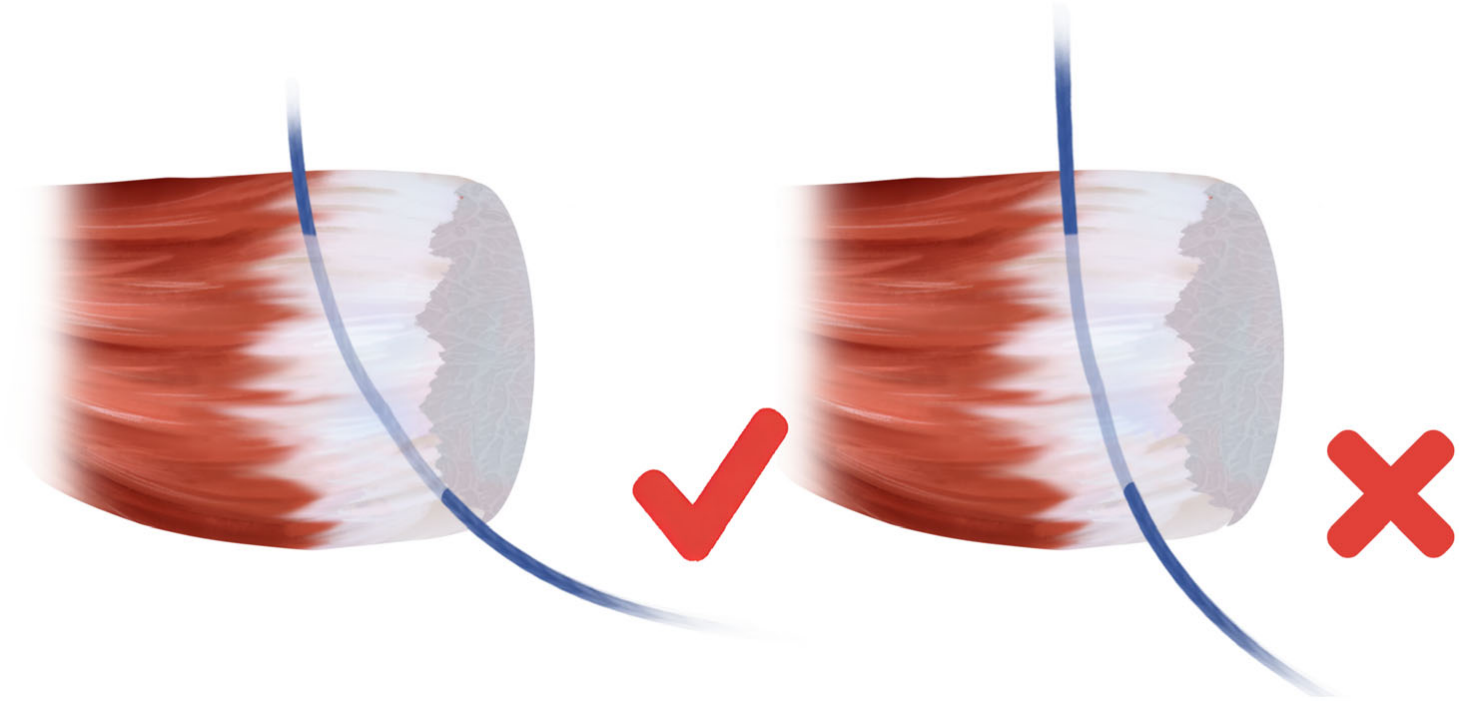
Optimal suture trajectory and entry geometry. Under lateral subacromial visualization, suture entry points are placed 2‐3 mm from the tear edge of the right rotator cuff, whereas exit points are positioned close to the margin. Excessively vertical trajectories are avoided to optimize tendon‐bone contact (laterality: right). Educational message: Shallow, margin‐proximal suture geometry enhances footprint compression and minimizes risk of tendon edge lift‐off.

**FIGURE 6:**
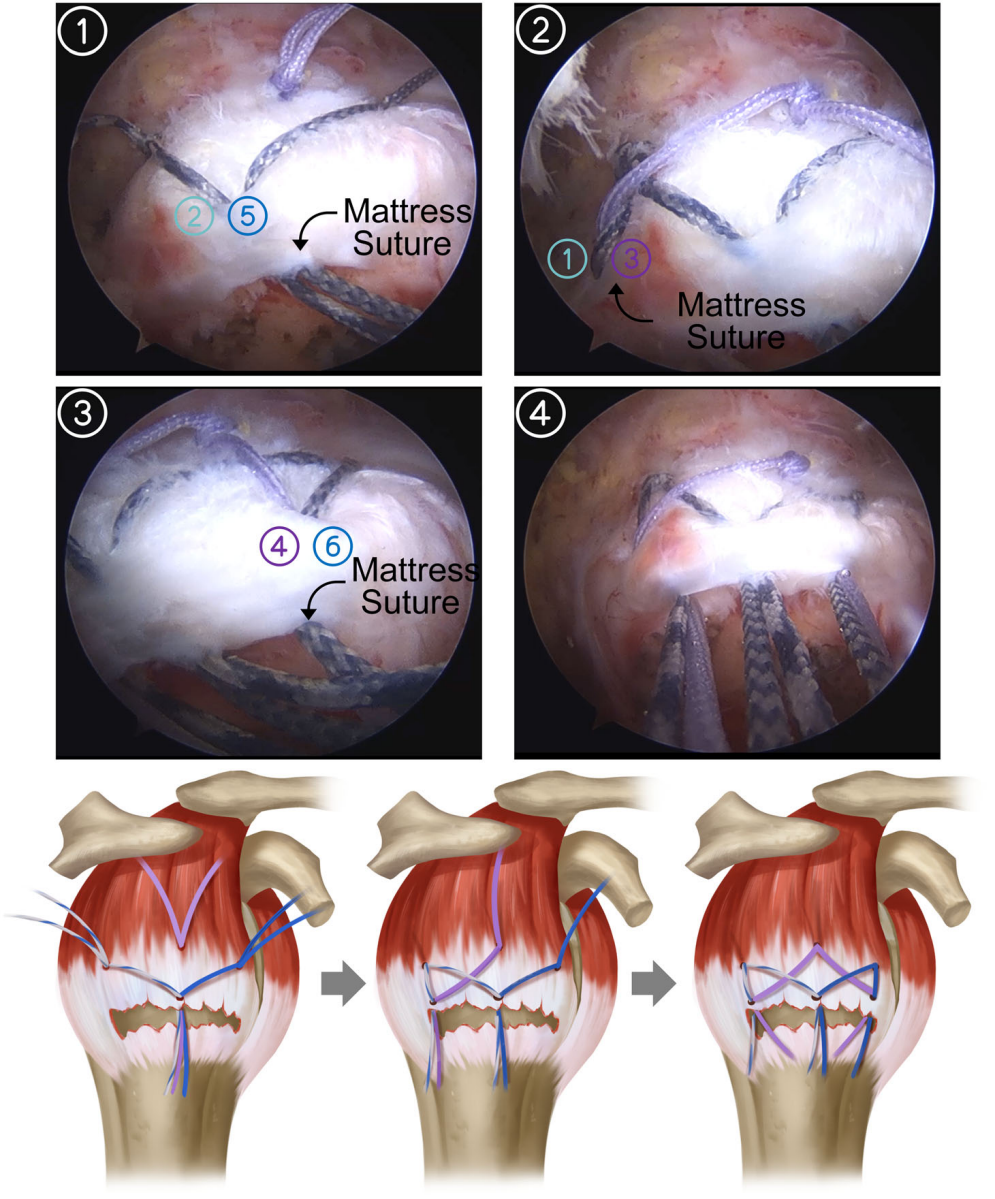
Diamond‐unit crisscross mattress reconstruction. From the lateral viewing portal (30° arthroscope), sutures are passed in a posterior‐to‐anterior crisscross pattern (1‐3, 2‐5, and 4‐6), forming interconnected diamond‐shaped units within the supraspinatus tendon. Final tightening achieves uniform tendon apposition onto the footprint (laterality: right). Educational message: The crisscross architecture converts discrete fixation points into a continuous load‐sharing network, improving biomechanical stability and footprint conformity.

Finally, all sutures are fixed to the lateral side of the greater tuberosity using a single lateral‐row anchor (ExtraLock Knotless PEEK Suture Anchor; Star Sports Medicine Co., Ltd., Beijing, China) (Figure 7). When tightening the sutures, it is crucial to note that sutures 3 and 4 should be tightened last to prevent excessive tension, which could potentially damage the rotator cuff tissue. The complete surgical sequence of the diamond‐unit crisscross mattress suture technique is shown in Video 1.

**FIGURE 7:**
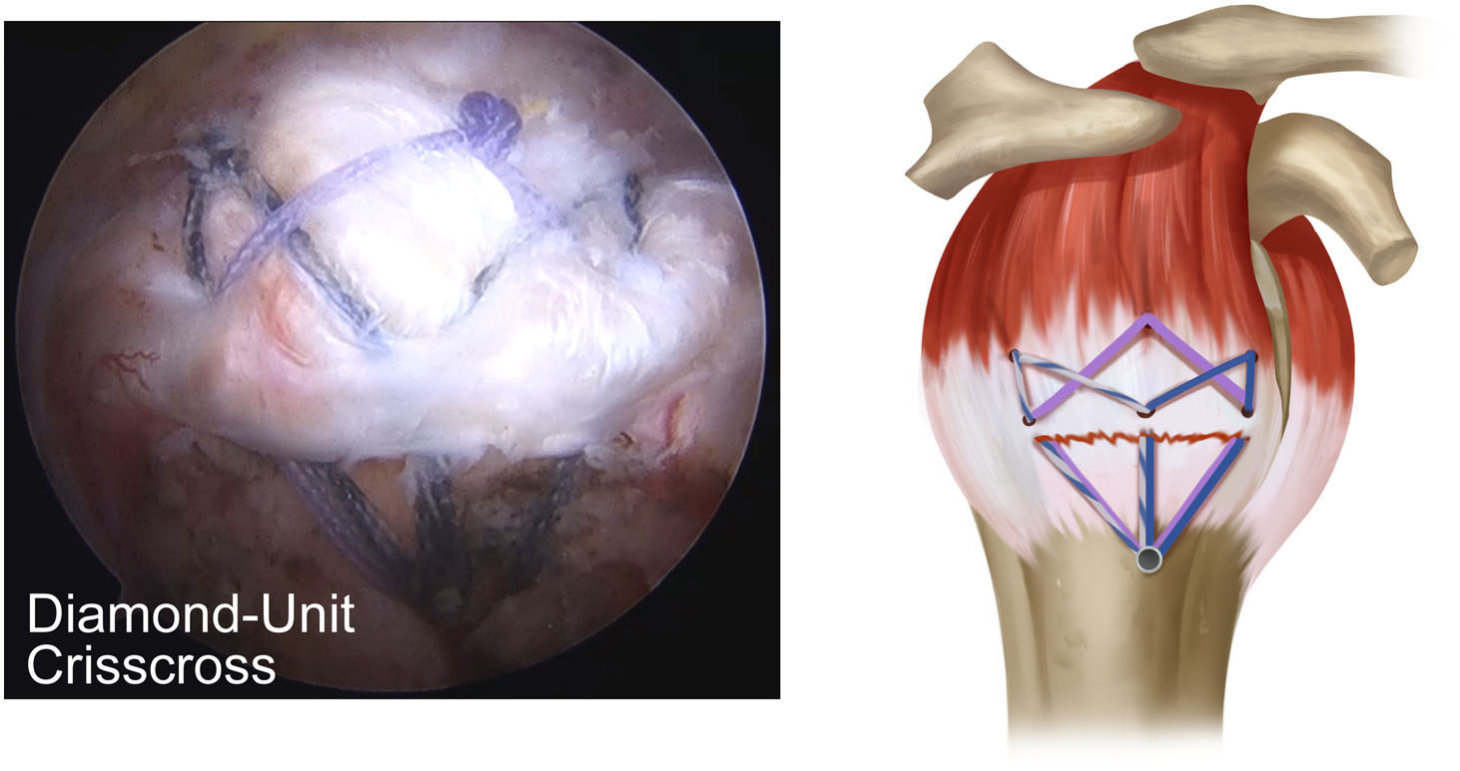
Final lateral‐row fixation. All suture limbs are secured to the lateral aspect of the greater tuberosity using a knotless lateral‐row anchor under lateral portal visualization in a beach‐chair position. Final construct shows complete tendon‐to‐bone apposition without residual gap formation (laterality: right). Educational message: A single lateral‐row anchor is sufficient when internal crisscross suture architecture provides primary load distribution and stability.

## DISCUSSION

The value of the diamond‐unit crisscross mattress configuration proposed in this article lies not simply in increasing footprint coverage but rather in altering the way tensile forces are transmitted within the torn tendon edge. After suturing and tying the rotator cuff, the sutures alternately cross from the inside and outside of the torn tendon edge, forming a series of interconnected diamond‐shaped units that are continuously distributed along the torn edge. This converts traditional point‐based fixation into a continuous surface coverage, enhancing the uniformity of tendon compression.

In conventional double‐row or suture‐bridge repairs, tensile loads are primarily transferred through a limited number of longitudinal suture limbs and concentrated at the lateral‐row anchors, making the construct vulnerable to collapse when lateral fixation fails.^11^, ^12^ In contrast, the present technique, based on knotted fixation at the medial‐row anchors, creates a series of continuous crisscross suture units within the tendon edge. This allows tensile forces to be dispersed through multiple internal load‐sharing pathways, thereby reducing reliance on the strength of any single anchor. This technique reshapes the tendon margin to enable a smooth, uniform fit against the bone surface, improving tendon‐to‐bone contact consistency and enhancing footprint coverage. By forming a continuous network of crisscross suture units, this method improves the distribution of tensile forces, further ensuring the stability of the repair while minimizing the risk of failure in the lateral fixation.

From a technical perspective, the stability achieved with a single lateral‐row anchor in this configuration is not dependent on the magnitude of lateral compression but rather on the intrinsic stability created by the suture structure within the tear margin. The sequential arrangement of diamond‐shaped units along the torn edge enables adjacent suture segments to constrain one another during loading, creating a state analogous to tension interlocking. Under these conditions, the lateral‐row anchor serves predominantly to maintain tendon‐bone apposition rather than acting as the primary load‐bearing element. Technical pearls and pitfalls are summarized in Table 1.

**TABLE 1:** Technical Pearls and Pitfalls

**Category**	**Technical Point**	**Description**
Pearl	Even distribution of sutures	All suture ends should be passed through the rotator cuff tissue and positioned more than 10 mm medial to the lateral edge of the tear to ensure even suture distribution. This helps achieve uniform tendon compression
	Insertion and exit point setup	The insertion points should be located approximately 2‐3 mm from the rotator cuff tear edge. The exit points should be placed as close as possible to the tear edge while avoiding an overly vertical entry angle. This increases the contact area between the tear edge and the bone surface and helps prevent edge lifting
	Suture tightening sequence	Sutures 3 and 4 should be tightened last to avoid excessive tension, which may damage the rotator cuff tissue
Pitfall	Uneven suture distribution	Uneven suture distribution may result in nonuniform compression, thereby compromising repair effectiveness and tendon‐to‐bone contact
	Incorrect entry and exit points	If the entry points are too far from the tear edge, suture distribution may become uneven, and tear coverage may be inadequate. If the exit points are too far from the tear edge or the entry angle is too vertical, the suturing effect may be impaired, and the tear edge may lift

This suture configuration also shows a distinct advantage in managing rotator cuff tears with irregular or degenerative margins. In clinical practice, crescent‐shaped tears or those with frayed edges often present challenges in achieving uniform reduction because of uneven tendon thickness and imbalanced tension distribution.^13^, ^14^, ^15^ Through repeated crossing and reversal of the suture path, the diamond‐unit crisscross structure progressively reshapes the tear margin, guiding it toward a flatter and more uniformly apposed interface with the bone surface. Additionally, by drawing the tear edges together, the configuration provides lateral stability. Importantly, this effect is achieved through geometric guidance of suture orientation rather than forceful traction, aligning with previous concepts aimed at reducing suture cut‐through and improving tissue‐holding capacity.^16^, ^17^

Rather than increasing anchor number or construct complexity, the present technique emphasizes improving repair efficiency through optimization of suture configuration. This approach directly addresses ongoing discussions in rotator cuff repair regarding the nonlinear relation between implant quantity and clinical benefit. For small‐ to medium‐sized tears, minimizing unnecessary anchor usage while preserving adequate footprint coverage may reduce surgical cost and avoid potential complications associated with multiple fixation points.^18^, ^19^, ^20^ Comparative advantages and disadvantages are detailed in Table 2.

**TABLE 2:** Technical Advantages and Disadvantages

**Category**	**Technical Point**	**Description**
Advantage	Reduced anchor usage	The use of a single lateral‐row anchor minimizes the number of implants required, which may reduce surgical cost and the risk of complications related to excessive anchor placement
	Uniform tendon compression	By incorporating both knotted and knotless fixation, this technique helps achieve uniform tendon compression across the footprint, thereby promoting tendon‐to‐bone contact and healing
	Enhanced stability	The crisscross suture configuration disperses tensile forces across multiple suture pathways, reducing reliance on any single anchor and improving the overall stability of the repair construct
	Improved tear‐margin conformity	The technique reshapes the tendon margin and allows it to conform more closely to the bone surface, which helps improve tendon‐to‐bone contact and minimize gap formation during healing
Disadvantage	Increased technical complexity	This technique may require greater skill and precision than simpler repair methods, particularly in suture placement and tension management. However, once surgeons become familiar with the procedure, the time required to perform the mattress suture can be reduced to less than 3 min
	Limited applicability in advanced cases	In cases of severe tendon degeneration or complex rotator cuff injuries, this method may not provide sufficient fixation, and additional anchors or alternative repair strategies may be required
	Not suitable for massive irreparable tears	This technique is mainly intended for small‐ to medium‐sized tears and is not recommended for massive irreparable tears, particularly those with Patte stage 3 retraction
	Need for further validation	Biomechanical validation of creep resistance and clinical outcome studies with at least 24 mo of follow‐up are still needed

min, minutes; mo, months.

Nevertheless, the applicability of this technique has defined limitations. In cases of massive tears, severe tendon retraction, or significantly compromised tissue quality (particularly at the tear margin), the crisscross configuration alone may be insufficient to achieve adequate reduction, and additional anchors or alternative repair strategies may still be required. The present study primarily focuses on the technical configuration and its theoretical rationale; biomechanical testing and clinical outcome studies are necessary to further validate the long‐term stability and healing potential of this construct.

Overall, the diamond‐unit crisscross mattress suture represents more than a minor modification of existing double‐row repair concepts. By redefining the geometric pathway of suture traversal within the torn tendon edge, this technique describes a suture‐configuration–centered mechanism for achieving construct stability in rotator cuff repair. While preserving procedural simplicity, it offers a perspective on reducing anchor dependence and improving tear‐margin conformity.

## DISCLOSURES

The authors (X‐H.W., C.F., G.Y., Y‐M.W., Q‐C.Z.) declare that they have no known competing financial interests or personal relationships that could have appeared to influence the work reported in this article.

## FUNDING

This work was supported by a grant from the Doctor Research Start‐up Fund (No. RC2024067).
